# Oral Health, Cognitive Function, and Mortality: Findings From National Surveys

**DOI:** 10.1093/geroni/igab046.790

**Published:** 2021-12-17

**Authors:** Bei Wu, Susie Keepper, Michele Saunders

## Abstract

Poor oral health, diabetes mellitus (DM), and cognitive impairment are common problems in older adults. Using national surveys, this symposium aims to present new findings regarding the impact of the co-occurrence of DM and poor oral health on cognitive function, cognitive decline, and mortality. This symposium will also cover the topic of dental care use among adult populations in the U.S. Using data from the Health and Retirement Study (HRS) (2006- 2018), the first study shows that adults with both DM and edentulism had the worst cognitive function, followed by those with edentulism alone, and those with DM alone. Using the same HRS data, the second study found that co-occurrence of DM and edentulism had a higher risk of more rapid cognitive decline with advancing age than the presence of each condition alone. The third study used data from the 2006-2016 HRS linked with mortality files, and revealed that the risk of diabetes and edentulism on mortality may vary across racial/ethnic groups. Using the Behavioral Risk Factor Surveillance System survey (2002-2018), the fourth study examined disparities of dental service utilization among racial/ethnic groups (Whites, Hispanics, Blacks, Asians, American Indians or Alaska Natives, and Native Hawaiian or other Pacific Islanders). Age differences in dental services were also compared between older adults and other younger and middle-aged populations. This symposium highlights the role of oral health in improving cognitive health. Policies and programs are needed to increase dental care access, a critical way to help maintain good oral health.

